# Digital PCR for Quantifying Norovirus in Oysters Implicated in Outbreaks, France

**DOI:** 10.3201/eid2212.160841

**Published:** 2016-12

**Authors:** David Polo, Julien Schaeffer, Nelly Fournet, Jean-Claude Le Saux, Sylvain Parnaudeau, Catherine McLeod, Françoise S. Le Guyader

**Affiliations:** Ifremer, Nantes, France (D. Polo, J. Schaeffer, J.-C. Le Saux, S. Parnaudeau, F.S. Le Guyader);; Santé Publique France, French National Public Health Agency, Saint-Maurice, France (N. Fournet);; Seafood Safety Assessment Ltd., Isle of Skye, Scotland, UK (C. McLeod)

**Keywords:** norovirus, digital PCR, France, shellfish, oysters, enteric infections, viruses, food safety

## Abstract

Using samples from oysters clearly implicated in human disease, we quantified norovirus levels by using digital PCR. Concentrations varied from 43 to 1,170 RNA copies/oyster. The analysis of frozen samples from the production area showed the presence of norovirus 2 weeks before consumption.

Shellfish have a long history as vectors of human enteric viruses; this relationship is particularly apparent with oysters and norovirus (*1*,*2*). Specific norovirus ligands found in oyster tissues facilitate the persistence of viral particles for several weeks, resistance to depuration, and strain selectivity by the oyster (*1*,*3*). Although advances have been made in virus detection in shellfish, quantification of norovirus in oysters associated with outbreaks still presents a challenge. More accurate quantification is essential for risk analysis and to understand the exact role played by shellfish in norovirus transmission, as this will be important to support the implementation of norovirus regulations. 

Norovirus reference materials are essential for quantification by real-time PCR, but they are not widely available for inclusion in standard curves; however, this limitation may be overcome by using digital PCR (dPCR) (*4*). This technology is based on partitioning of the sample into thousands of individual PCRs that contain, in theory, 1 or no copies of the nucleic acid target. After amplification, the total number of target molecules is calculated, with no need for external reference standards (*4*). The partitioning of samples into large numbers of subsamples may also decrease the impact of enzyme inhibitors possibly linked to matrix-type components. This partitioning may be particularly advantageous for the detection of viruses in food and environmental samples, which tend to be complex, with a large variety of inhibitory compounds but relatively low numbers of viruses (*4*,*5*). Norovirus-specific primers and probes targeting the open reading frame 1–2 region used for the real-time reverse transcription PCR were used in a microfluidic-based dPCR to enable norovirus quantification in oyster samples associated with outbreaks.

## The Study

In France, medical doctors who diagnose norovirus gastroenteritis in ≥2 persons who shared a common meal are required to declare a suspected foodborne illness outbreak. All meal participants then receive a standardized questionnaire that addresses the foods consumed, the symptoms, and the timing of illness, allowing the calculation of the relative risk and its 95% CI. Information on outbreaks to laboratories must be transmitted quickly to enable collection of samples that are directly linked to the clinical cases.

Eight outbreaks were considered for this study on the basis of the following criteria: clinical diagnosis of norovirus in sick consumers; epidemiologic confirmation that oysters were implicated; and rapid notification of responsible oyster production areas. The outbreaks occurred during the winter months in private houses except for 1 that occurred in a nursing home (outbreak 8) (Table). The attack rate varied from 43% to 100%, with median incubation times between 0.5 and 2 days. Fecal samples available for 2 outbreaks (1 for outbreak 6 and 3 for outbreak 8) confirmed the presence of norovirus (National Reference Center for Enteric Viruses, Dijon, France, pers. comm.). Eight shellfish samples were collected from batches that were directly implicated, and 1 sample was taken from leftovers in the nursing home’s refrigerator, increasing the likelihood that the samples were representative of consumed oysters. An additional 16 samples were collected from implicated production areas located along different coasts of France, including frozen samples (during the winter and spring months, Ifremer laboratories doing official control monitoring of shellfish for *Escherichia coli* routinely freeze leftover samples). Viruses were eluted from oyster digestive tissues by using the reference method (*6*), and then quantified using the QuantStudio 3D Digital PCR system (Thermo Fisher, Villebon, France) (Technical Appendix).

**Table:** Characteristics of outbreaks of norovirus infection associated with consumption of oysters, analyzed oyster samples, and virus concentrations obtained by digital PCR*

Outbreak no.	Epidemiology information			Sample no.	Samples analyzed			Viral RNA copies/oyster	
Date of consumption	No. sick/ no. exposed	Days to illness onset†	Days to sampling‡	Sample location§	Genogroup GI	Genogroup GII
1	2014 Feb 23	7/16	0.5	3486	4	Same batch		ND	1.09 × 10^2^
				3488	7	Prod area		ND	ND
				3489	7	Prod area		ND	2.96 × 10^2^
2	2014 Feb 27	3/4	2	3498	5	Same batch		ND	3.72 × 10^2^
3	2014 Mar 16	4/4	1	3519	2	Prod area		ND	2.02 × 10^2^
				3517	5	Prod area		ND	6.81 × 10^2^
				3518	2	Prod area		3.80 × 10^2^	6.15 × 10^2^
				3531	15	Prod area		1.08 × 10^3^	ND
				3532	15	Prod area		3.88 × 10^2^	ND
4	2014 Dec 12	3/3	No data	3703	6	Same batch		1.18 × 10^2^	ND
				3694	–4	Prod area		ND	1.70 × 10^2^
5	2014 Dec 14	2/2	1.5	3704	3	Same batch		ND	1.21 × 10^2^
				3705	3	Same batch		2.74 × 10^2^	44.1
				3695	–6	Prod area		ND	43.2
				3698	–6	Prod area		1.18 × 10^2^	ND
				3700	–6	Prod area		1.1 × 10^3^	9.50 × 10^2^
6	2014 Dec 27	3/6	0.5	3733	6	Same batch		ND	1.26 × 10^2^
7	2015 Jan 9	3/4	1.5	3740	3	Same batch		9.20 × 10^2^	ND
				3738	–3	Prod area		1.17 × 10^3^	ND
				3739	–3	Prod area		6.38 × 10^2^	53.4
8	2015 Mar 29	16/36	2	3816	3	Consumed		ND	82.1
				3817	3	Same batch		1.85 × 10^2^	ND
				3791	–19	Prod area		ND	1.87 × 10^2^
				3792	–19	Prod area		8.28 × 10^2^	ND
				3822	10	Prod area		1.28 × 10^2^	ND

*ND, not detected; Prod, production. †Median days to onset of vomiting or diarrhea.  ‡Days from date of consumption to sample collection. §Same batch = samples from batch of oysters consumed.

No norovirus was detected in 1 oyster sample; norovirus genogroups GI, GII, or both were detected in 9, 11, and 4 samples, respectively (Table). Overall, norovirus concentrations ranged from 43 to 1,170 RNA copies/oyster; the highest concentrations detected were GI. For outbreak 8, in which a leftover sample from the implicated meal was obtained, norovirus GII was detected at a concentration of 82 RNA copies/oyster, whereas norovirus GI was detected at a concentration of 185 RNA copies/oyster in the same batch collected from the oyster farm. 

In a previous dose–response model for norovirus GI and GII based on outbreak investigations, differences were observed between consumers with the secretor phenotype, for which infection and disease probability were high at low doses compared with nonsecretor phenotypes (*7*). Although method sensitivity may need to be improved, the concentrations reported here are consistent with observed illness in dose–response studies to date (*8*). Norovirus GI and GII were detected in oyster samples from the production area and in 4 fecal samples (National Reference Center for Enteric Viruses, pers. comm.). 

Because oyster contamination occurs through the filtration of seawater contaminated by human sewage, many contamination events involving both norovirus genogroups and different strains have been described worldwide; this study provides additional evidence of the diversity of contamination (*1*). In contrast to person-to-person transmission in which GII strains dominate, oysters favor the transmission of some specific GI strains, a major consideration for the global epidemiology of norovirus (*1*,*3*). Thus, identifying if oysters implicated in outbreaks are contaminated with norovirus GI or GII is important, because genetic susceptibility means that some consumers do not become infected with certain GI or GII strains; this affects the disease and favors the distribution of some norovirus strains. Such a comprehensive approach will provide information for risk analysis and assist in understanding norovirus infections (*7*,*9*).

Although we obtained some norovirus sequences from 6 implicated batches, confirming the specificity of the dPCR, we believe that the development of technology such as next-generation sequencing will provide more detailed information on the full range of strains present in samples. Obtaining more accurate information on strain diversity and quantification will be valuable for molecular epidemiology studies and management.

In France, oysters are a popular dish, especially during December–April, when they are in the optimal low-fat condition for consumption. They are opened just before consumption and eaten raw; intravalvular seawater is tipped out, thus eliminating food handler contamination. Because this is the highest period for potential contamination by norovirus, samples are kept frozen by laboratories in France for analysis in case of outbreaks. In the current case, this was useful because it demonstrated the presence of norovirus up to 19 days before the shellfish were marketed. This detection in samples collected 2 weeks before an outbreak suggests that illness could have been prevented. Control shellfish samples from different production areas were analyzed at the same time and were negative for norovirus (data not shown), correlating well with the estimated NoV prevalence of less than 10% in France (*10*).

## Conclusions

This study demonstrates that outbreaks could be prevented by performing shellfish analysis at times of the year at which norovirus risk is elevated, such as the winter season, and following microbial alert events such as sewage overflows and heavy rainfall. Application of dPCR to shellfish implicated in outbreaks will provide accurate quantification, which is useful for further risk analysis studies. This application will help to improve regulations and enhance the safety of products on the market, keeping in mind that the sanitary quality of coastal areas is of primary concern.

Technical Appendix Sample analysis, amplification conditions, quantification, and typing of oysters implicated in norovirus outbreak, France.
