# Supplementary material for: Digital PCR for Quantifying Norovirus in Oysters Implicated in Outbreaks, France
Source: Emerg Infect Dis. 2016 Dec;22(12):2189–91. doi: 10.3201/eid2212.160841 (PMC5189157; doi:10.3201/eid2212.160841)
Supplement: Technical Appendix — Sample analysis, amplification conditions, quantification, and typing of oysters implicated in norovirus outbreak, France. [file 16-0841-Techapp-s1.pdf]

Article DOI: http://dx.doi.org/10.3201/eid2212.160841

Digital PCR for Quantifying Norovirus in Oysters Implicated in Outbreaks, France

Technical Appendix

Oyster Sample Analysis

Oyster samples were sent to the laboratory by the veterinary service for the leftover sample and the 8 batch samples, and by Ifremer technicians for the 16 samples collected from the implicated production areas. Oysters, maintained at 4°C during shipment (or frozen for the 8 samples collected before outbreaks occurred), were washed, shucked, and immediately dissected to recover the digestive tissues. Viruses were eluted from 2 g of digestive tissues by using the proteinase K protocol and nucleic acids (NAs) were extracted by using the NucliSens kit (bioMérieux, Lyon, France) (*1*). Extraction efficiency was verified using with Mengovirus added to the tissue as specified in the reference method.

Amplification Conditions

Mengovirus amplification was performed using primers, probe, and cycling conditions described in the reference method (*1*), and the extraction efficiency was calculated for each sample. As specified in the reference method, only samples with an extraction efficiency above 1% were considered acceptable.

For norovirus detection, primers and probes targeting the open reading frame 1–2 region were used. The sequences were as follows: For GI, QNIF4 (FW) (5′-CGC TGG ATG CGN TTC CAT-3′ with N: A/C/G/T), NV1LCR (REV) (5′-CCT TAG ACG CCA TCA TCA TTT AC-3′) and the probe NVGG1p (5′-TGG ACA GGA GAY CGC RAT CT-3′ with Y: C/T and R: A/G). For GII, QNIF2 (FW) (5′- ATG TTC AGR TGG ATG AGR TTC TCW GA-3′ with R: A/G and W: A/T), COG2R (REV) (5′-TCG ACG CCA TCT TCA TTC ACA-3′), and the probe QNIFs (5′-AGC ACG TGG GAG GGC GAT CG-3′). Probes were labeled with ZEN-Iowa BlackFQ double-quenched (*2*).

cDNA was obtained using the SuperScript III Reverse Transcription System (Invitrogen Thermo Fisher, Villebon, France). Reverse transcription conditions were 30 min at 55°C and 15

min at 70°C. Five µL of cDNA was then amplified with the QuantStudio 3D Digital PCR Master Mix (Invitrogen Thermo Fisher), using the primers and probes at the concentrations recommended for the real-time reverse transcription PCR (rRT-PCR) (ISO/DIS 15216). Samples were loaded onto the QuantStudio 3D Digital PCR 20K chips with 20,000 partitions of 865 pL each, and then loaded into the QuantStudio 3D Digital PCR system (Thermo Fisher, France). After 10 min at 96°C, 45 amplification cycles were performed.

Quantification

Following amplification, endpoint fluorescence of each partition was analyzed with the QuantStudio 3D AnalysisSuite Cloud Software (version 3.0.3; Invitrogen Thermo Fisher) to statistically estimate the number of copies of target DNA. Final quantification data were provided by the software through counting the number of positive chambers (H) out of the total number of chambers (C) per chip. Then, the Poisson distribution was used to estimate the average number of molecules per partition (λ), so $\lambda = -\ln(1 - H/C)$. A no-template control consisting of water instead of NA extract was included in each run. The final result is expressed as cDNA copies per microliter.

Concentration per Oyster

The concentration per oyster was back-calculated using an efficiency of 100% for cDNA production (checked by real-time RT-PCR, data not shown), and the volume of NA extract analyzed. First, the NoV concentration was calculated per g of DT, and then per oyster based on the total weight of one animal.

Typing

Positive samples that were collected from batches implicated in the outbreaks were sequenced (except three samples due to lack of material). NA extracts were amplified by the standard RT-PCR method with the same reverse transcription and platinum *Taq* polymeraseenzymes. Primers targeting the polymerase and the capsid regions were used in a 2-step semi-nested format, and 40 cycles of amplification were performed (using dedicated rooms and all precautions to avoid cross-contamination). Amplicons from positive samples were purified and sequenced directly (*3*).

References

1. ISO/TS 15216–1. Microbiology of food and animal feed -horizontal method for determinaion of hepatitis A virus and norovirus in food using real-time RT-PCR, Part 1: method for quantification. 2013.

2. Ishii S, Kitamura G, Segawa T, Kobayashi A, Miura T, Sano D, et al. Microfluidic quantitative PCR for simultaneous quantification of multiple viruses in environmental water samples. Appl Environ Microbiol. 2014;80:7505–11. http://dx.doi.org/10.1128/AEM.02578-14

3. Le Guyader FS, Le Saux J-C, Ambert-Balay K, Krol J, Serais O, Parnaudeau S, et al. Aichi virus, norovirus, astrovirus, enterovirus, and rotavirus involved in clinical cases from a French oyster-related gastroenteritis outbreak. J Clin Microbiol. 2008;46:4011–7. http://dx.doi.org/10.1128/JCM.01044-08

Technical Appendix Table. Norovirus typing in samples collected in some batches implicated in outbreaks, France*

Sample	NoV GI	NoV GII
3498		GII.6 (cap)
3704		GII.17 (pol)
3705		GII.3 (cap)
3733		GII.4 Syd (cap)
3740	GI.5 (cap)	
3817	GI.4 (cap)	

*NoV sequence was confirmed after amplification of a fragment targeting the polymerase (pol) or capsid (cap) region.
